# IgA-dominant postinfectious glomerulonephritis: a case report

**DOI:** 10.3389/fneph.2023.1284814

**Published:** 2023-11-03

**Authors:** Rodolfo Moreno-Alvarado, Guillermo Navarro-Blackaller, Werner De Leon-Pérez, David Armas-Eguizabal, Jonathan Chávez-Iñiguez

**Affiliations:** ^1^ Nephrology Service, Military Medical Center, Guatemala, Guatemala; ^2^ Nephrology Service, Civil Hospital of Guadalajara, Guadalajara, Jalisco, Mexico; ^3^ SERPAT, Private Pathology Laboratory, Guatemala, Guatemala; ^4^ Internal Medicine Resident, Military Medical Center, Guatemala, Guatemala

**Keywords:** postinfectious glomerulonephritis, immunoglobulin A, acute kidney injury, kidney biopsy, case report

## Abstract

**Introduction:**

Acute postinfectious glomerulonephritis (APIGN) is an immunological glomerular disease that is an important health issue in developing countries. The incidence remains high in developing countries with a male-to-female ratio of 2:1 and age predominantly above 50 years. In this case study, we present a patient with a history of *Staphylococcus epidermidis* infection, a past medical history of diabetes mellitus, and histopathological findings of APIGN with Immunoglobulin A (IgA) deposition.

**Methods:**

A 58-year-old male presented to the emergency room with a 6-day history of severe low back pain. Three days later, the patient developed fever, chills, abdominal pain in the upper quadrant and a subsequent lower limb cellulitis. Various immunological tests, imaging studies, and kidney biopsy were performed to arrive at a diagnosis.

**Results:**

Following the diagnosis and treatment of Cholangitis and *Staphylococcus epidermidis*, further investigation led to a diagnosis of IgA-dominant APIGN. IgA-dominant APIGN was treated with antibiotics, renin-angiotensin-aldosterone system inhibitors and steroids, and the patient was discharged from the hospital.

**Conclusion:**

In developing countries, APIGN is a relatively common presentation of kidney damage due to acute kidney injury and nephritic syndrome. IgA-dominant APIGN is a rare but increasingly recognized morphological variant in which IgA is the sole or dominant immunoglobulin. This unique presentation and multidisciplinary approach for diagnosing and treating IgA-dominant APIGN need to be considered and understood by healthcare professionals to better help these patients. Further investigation is needed to understand the best treatment of this IgA-dominant APIGN presentation and its prognosis.

## 1 Introduction

Acute postinfectious glomerulonephritis (APIGN) is an immunological glomerular disease that is an important health issue in developing countries. The incidence remains high in developing countries with a male-to-female ratio of 2:1 and age predominantly above 50 years. The incidence of APIGN has declined in industrialized countries. Histopathologic findings usually consist of diffuse cellular proliferation and exudate of neutrophils and monocytes associated with granular deposits of complement C3, Immunoglobulin G, and occasionally Immunoglobulin M in immunofluorescence. Less commonly, Immunoglobulin A (IgA) deposits may be present (1, 2). In this case study, we present a patient with a history of *Staphylococcus epidermidis* infection, a past medical history of diabetes mellitus, and histopathological findings of APIGN with IgA deposition confirming a worse prognosis than primary IgA nephropathy (3–5).

## 2 Case study

A 58-year-old male with a 40-year history of alcoholism, 5 years of sobriety and type 2 diabetes mellitus presented to the emergency room. No other medical history was pertinent except for a 6-day history of severe low back pain that required hospitalization. Three days later, the patient developed fever, chills, and abdominal pain in the right upper quadrant, associated with jaundice and lower limb edema. On admission to the emergency room, laboratory tests (**Table 1**) showed evidence of leukocytosis related to abdominal pain and jaundice. The patient was diagnosed with Cholangitis, and antibiotic treatment with ampicillin-sulbactam was initiated. Hepatic and biliary tract ultrasonography revealed cholelithiasis without intra- or extra-hepatic biliary tract dilation; therefore, surgical treatment was not required. The initial lower limb edema increased with changes in color and temperature in the right lower limb, for which cellulitis was considered. Clindamycin 600 mg IV every 8 h was associated with clinical improvement after 48 h with resolution of fever. Blood cultures were performed and were positive for *Staphylococcus epidermidis*. Furthermore, acute kidney injury and hematuria were observed; therefore, focus was placed on ruling out glomerulonephritis. Considering nephritic syndrome, autoimmune tests were requested, all of which were negative. Ultrasonography showed bilateral normal sized kidneys. The results of these laboratory tests are summarized in **Table 1**.

**Table 1:** Laboratory values.

LABORATORIES						
	Admission	Nephrology Consult	Reference		Neprhology Consult	Reference
Glucose (mg/dl)	85	91.8	74 ―115	Serum albumin (gr/dl)	2.78	3.5―4.5
Creatinine (mg/dl)	0.63	1.53	0.61―1.3	Total cholesterol (mg/dl)	184	<200 low risk
BUN (mg/dl)	30	22	6.0 ― 26.0	LDL cholesterol (mg/dl)	128	<130/DM <70
Sodium (meq/L)	139.5	138	136 ―145	Triglycerides (mg/dl)	165	< 150
Potasium (meq/L)	4.05	3.94	3.5 ―5.1	NT-proBNP (pg/ml)	260	<100
Calcium (mg/dl)	7.51	7.89	8.6 ―10.3	24 h Protein (gr/day)	0.58	0.05-0.15
Phosphorus (mg/dl)	3.68	4.81	2.5―4.6	**Urinalysis**		
Magnesium (mg/dl)	1.95	1.75	1.60―2.6	protein (mg/dl)	30	neg
Chloride (meq/L)	105	105	98―111	White blood cells (hpf)	15	0―5
Total Bilirrubin (mg/dl)	2.12	0.59	0.3―1.2	Red blood cells (hpf)	>35	1-5cells
Direct Bilirrubin (mg/dl)	1.03	0.23	0.0―0.5	Acanthocytes	80%	
Indirect Bilirrubin (mg/dl)	1.09	0.37	0.10―0.60			
White Blood Cell (10∧3/uL)	19	7.45	5―10			
Hemoglobin (g/dl)	8	7.5	12―18			
Hematocrit (%)	26.89	24	38―48			
Platelets (10∧3/uL)	565	380	150―450			
Erythrocyte sedimentation rate (mm/hr)	127	140	0.00―20			
Prothrombin time (seg)	12.3	12.3	9.4―12.5			
Partial thromboplastin time (seg)	20.5	20.5	25.4―36.9			
Direct COOMBS test		positive	negative			
CK TOTAL (U/I)		12.2	< 190			
VDRL		Neg	neg			
Complement C3 (gr/L)		0.327	0.841―1.67			
Complement C4 (gr/L)		0.28	0.164―0.31			
FANA		neg	neg			
Antiestreptolisin (UI/ml)		<200	<200			
Immunoglobulin IgG (gr/L)		3.05	8.0―18			
Immunoglobulin IgA (gr/L)		0.475	0.8―3.87			
ANCA PR3-MPO		neg	neg			
HIV		neg	neg			
Hepatitis C virus		neg	neg			
Hepatitis B virus		Neg	neg			

**Figure 1:**
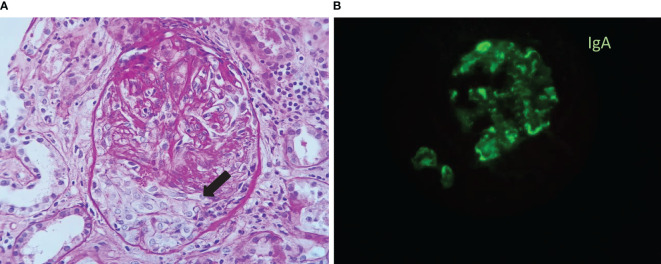
**(A)** Periodic-acid Schiff (PAS) stain: Glomerulus with a cellular crescent (black arrow). **(B)** Immunofluorescence (IF): focal positivity for IgA with a granular deposition along the glomerular basement membrane in a “garland” pattern.

A kidney biopsy revealed 20 glomeruli with mesangial expansion, irregularity in the glomerular basement membrane, endocapillary hypercellularity (7 glomeruli), and extra-capillary hypercellularity (4 glomeruli). Tubulo-interstitium with edema and mild lymphocytic infiltration were also observed. Granular and erythrocyte casts with a few epithelial cells were observed in the tubular lumen. Tubulitis (+), no tubular atrophy or interstitial fibrosis. Immunofluorescence with: IgG, IgM, Kappa, Lambda (-); IgA and C3 positive (++) focal in the glomerular basement membrane with a “garland pattern” confirmed the diagnosis of acute postinfectious glomerulonephritis (APIGN) with IgA dominance (**Figures 1A, B**). Electron microscopy was not performed because was not available at our facility.

Considering the histopathological pattern of crescents and the rapidly progressive rise of creatinine, persistence of hematuria, and proteinuria, we decided to treat the patient with intravenous methylprednisolone pulses (500mg) for 3 days. Then we switched to oral prednisone (1 mg/kg/day), tapering the dose progressively until suspension after 4 weeks. The infection was resolved, and the patient was discharged from the hospital with outpatient follow-up, where a 50% decrease in proteinuria was observed compared to baseline; the hematuria was resolved, but the elevation of creatinine (2.09 mg/dl) remained without returning to baseline values.

## 3 Discussion

In developing countries, APIGN is a relatively common presentation of kidney damage due to acute kidney injury and nephritic syndrome. IgA-dominant APIGN is an increasingly recognized morphological variant in which IgA is the sole or dominant immunoglobulin. APIGN is usually reported in patients with infections secondary to streptococci, but it is important to raise awareness that staphylococcus as well as gram-negative bacteria has been increasing in more recent reports with S. aureus being the most common agent in IgA-dominant APIG. The pathogenic mechanism of selective IgA deposition in patients with staphylococcal infections is not completely understood but it is likely to be a pathogen-host interaction as well as the staphylococcal enterotoxin that can act like a superantigen in the case of S. aureus but it does not explain it in the case of coagulase-negative staphylococci (S. epidermidis) that do not produce enterotoxin, this raises the suspicion of the presence of other specific surface antigens of each staphylococcus (6).

The clinical manifestations range from nephritic syndrome to asymptomatic glomerulonephritis (1). Kidney biopsy remains the gold standard for diagnosis because, to the best of our knowledge, no serological markers have been found to be pathognomonic or diagnostic for the disease. Among the histopathologic findings of IgA-dominant APIGN secondary to Staphylococcus, it may vary depending on the variant of the microorganism to which it is associated: acute proliferative pattern similar to that seen in post streptococcal glomerulonephritis in patients with infection associated with Staphylococcus aureus, usually in diabetic patients, with alcohol consumption or neoplasms, and a pattern of type 1 membranoproliferative glomerulonephritis in cases secondary to Staphylococcus epidermidis infection, usually in patients with ventriculo-vascular shunts. Another finding that can guide IgA-dominant APIGN is the C4 staining in the renal biopsy, since it is frequently negative in primary IgA nephropathy due to the activation of the alternative complement pathway. The reason why different histopathological patterns occur and the lower incidence of S. epidermidis is unknown and more studies are needed (2–7). For a favorable evolution are: the absence of glomerulosclerosis due to diabetes mellitus, young age, female sex, immunocompetence, and low creatinine levels at the time of biopsy. Presenting with glomerulosclerosis secondary to diabetes mellitus and older age were the worst prognostic indicators because > 80% of the patients progressed to end-stage kidney disease (3). IgA-dominant APIGN can present with hypocomplementemia, and elevated serum IgA levels may be involved in its pathogenesis.

Huang et al. in a retrospective and observational single-center study of 50 patients in 2020 reported acute endocapillary proliferative glomerulonephritis and exudative glomerulonephritis as the most common histological pattern of IgA-dominant APIGN and higher proportion of crescents compared to primary IgA nephropathy. For this reason, we considered primary IgA nephropathy unlikely considering the histopathological changes were not characteristic, and our patient was diagnosed with IgA-dominant APIGN due to the findings of: endocapillary proliferation, crescents and presence of IgA and C3 with the same intensity in glomerular basement membrane in IF associated with risk factors of diabetes mellitus, alcoholism and recent infection (8).

Usually, treating IgA-dominant APIGN is supportive, with treatment focus on treating the triggering infectious process. In some cases, such as those related to Staphylococcus aureus, brucellosis, and schistosomiasis, it can progress despite the eradication of the infection.

Previous reports of IgA-dominant APIGN risk factors for progression to kidney failure are: estimated glomerular filtration rate (eGFR) < 30 ml/min/1.73 m^2^ at presentation, moderate to severe interstitial fibrosis, tubular atrophy, and non-treatment with renin-angiotensin-aldosterone system blockers (6). In these cases, considering the cascade of glomerular inflammation, treatment with steroids, cytotoxic agents, and immunosuppression has been considered (1, 9), but no randomized clinical trials can validate them.

## 4 Conclusions

APIGN are secondary to infection caused by both gram-positive and gram-negative bacteria. IgA-dominant APIGN variant has been increasing and usually occurs in Staphylococcus aureus infection and less frequently with Staphylococcus epidermidis. The differential diagnosis from other similar diseases depends on clinical and pathological features. Elevated serum IgA levels may be involved in its pathogenesis. Treatment is based on the eradication of the infection with antibiotics.

In the present case, despite having been treated with antibiotics, the patient´s clinical picture did not improve, the kidney damage did not resolve nor improve as expected; when kidney biopsy showed crescents we decided to start steroids considering the severe inflammation pattern. IgA-dominant APIGN has a worse prognosis than traditional APIGN and primary IgA, which may be related to stronger inflammatory reaction caused by infection-mediated IgA deposition. Further investigation is needed to understand the best treatment of this IgA-dominant APIGN presentation and its prognosis.

## Data availability statement

The original contributions presented in the study are included in the article/supplementary material. Further inquiries can be directed to the corresponding author.

## Ethics statement

Written informed consent was obtained from the individual(s) for the publication of any potentially identifiable images or data included in this article.

## Author contributions

RM-A: Writing – original draft. GN-B: Writing – review & editing. WDL-P: Writing – review & editing. DA-E: Writing – review & editing. JC-I: Writing – review & editing.
